# Germline mutations of* GCM2* cause a novel variant of hereditary primary hyperparathyroidism

**DOI:** 10.1007/s13304-025-02179-0

**Published:** 2025-05-06

**Authors:** Maurizio Iacobone, Sara Watutantrige-Fernando, Stefania Zovato, Silvia Tognazzo, Silvia Dughiero, Veronica Augenti, Valentina Camozzi, Caterina Mian, Francesca Torresan, Claire Nomine-Criqui, Laurent Brunaud

**Affiliations:** 1 Endocrine Surgery Unit, Department of Surgery, Oncology and Gastroenterology, University of Padova, Padua, Italy; 2 Cancer Family Clinic, Veneto Institute of Oncology IOV-IRCCS, Padua, Italy; 3 Endocrinology Unit, Department of Medicine, University of Padova, Padua, Italy; 4 Department of Gastrointestinal, Visceral, Metabolic, and Cancer Surgery (CVMC), INSERM N-GERE, Université de Lorraine, CHRU Nancy, Nancy, France

**Keywords:** Hereditary hyperparathyroidism, *GCM2*, Parathyroidectomy, Primary hyperparathyroidism

## Abstract

Primary hyperparathyroidism (pHPT) occurs as hereditary disease in approximately 10% of cases. *GCM2* germline mutations have been recently described as responsible for the development of a novel variant of hereditary pHPT. This study aimed to determine the features of *GCM2*-related pHPT. Demographics, laboratory, and surgical data were assessed in a series of 17 index cases carrying *GCM2* mutations undergoing surgery for pHPT. The *GCM2* germline pathogenic variant c.1181 A>C p.(Tyr394Ser) was detected in 59% of cases. *GCM2*-related pHPT was diagnosed at a median age of 57 years (range 32–82) with a Female/Male ratio 1.8. Preoperative median calcemia was 2.89 mmol/L (range 2.69–3.8). Family history of pHPT was absent in 65% of cases. Complete clinical, surgical and follow-up data were available for 13 patients. At initial surgery, bilateral neck exploration with subtotal parathyroidectomy was performed in 46% of patients; achieving cure in all cases at a median follow-up of 51 months (range 7–60). In the remaining cases undergoing selective parathyroidectomy, a persistent pHPT occurred in 3 cases; recurrent pHPT in 1 patient (after a disease-free interval of 4 years) while 3 are disease free at a mean follow-up of 21 months. Thus, at an overall prolonged follow-up (median 48 months, range 7–216), multiglandular involvement occurred in 77% of cases. *GCM2* germline mutations may cause hereditary pHPT, even if it may mimic sporadic variant due to the absence of familial history and late onset. The main feature is multiglandular involvement, needing bilateral neck exploration and subtotal parathyroidectomy to achieve long-term cure.

## Introduction

Primary hyperparathyroidism (pHPT) is a common endocrine disorder, which occurs as a sporadic disease in 90% of cases, with a peak incidence between the age of 60 and 70 in women, caused by a single parathyroid adenoma in 90% of cases, with extremely high rate of cure at long term after selective parathyroidectomy. Conversely, pHPT may occur in 10% of cases as a hereditary disease; in this setting, usually, it presents in a familial context, at an earlier age, with a frequent multiglandular parathyroid involvement, causing reduced cure rates after selective parathyroidectomy and subsequently requiring iterative surgery [1].

Hereditary pHPT may be associated to other endocrine or non-endocrine neoplasms, as a part of syndromic disease, as occurs in case of multiple endocrine neoplasia (MEN) type 1, 2 A or 4 and hyperparathyroidism-jaw tumor (HPT-JT) Syndrome (caused by mutations in *MEN1, RET, CDKN, CDC73* genes, respectively), or as familial isolated hyperparathyroidism (FIHPT), without any association with further syndromic features [1–3].

The genetic basis of FIHPT is largely unknown; it may be caused by several and sometimes still unknown genes, representing either a stand-alone non-syndromic entity, or it may represent an incomplete expression of a syndromic variant of hereditary pHPT [1].

In recent years, activating mutations in *GCM2* (*Glial cell missing 2*) gene have been suggested as a possible novel cause of FIHPT [4], but, due to the rarity of the disease, very few data are available.

Due to the peculiar features of hereditary pHPT, the correct and early identification of the disease is fundamental to plan the extent and timing of surgery, and to adopt a tailored approach.

The present paper aimed to present the genetic, biochemical, clinical, pathological features and the outcomes of surgery in a novel series of patients undergoing surgery for *GCM2*-related pHPT and revise the available literature on this topic.

## Methods

The present retrospective study focused on 17 unrelated index-case patients undergoing surgery for *GCM2*-related pHPT at Endocrine Surgery Unit, University of Padua, Italy (*n* = 11), and Endocrine Surgery Unit, University of Nancy, France (*n* = 6), between 2006 and 2024.

Inpatient and outpatient medical records were retrospectively reviewed, focusing on genetic, demographics, anamnestic, laboratory values (including serum calcium, intact PTH, daily urinary calcium levels), surgical details (including the extent of neck exploration and parathyroid removal), pathological data, and outcomes. Follow-up data were finally further updated by telephone interview with the patient and/or the general practitioner/referral physician. Patients without outcomes data or a postoperative follow-up less than 6 months were excluded from outcomes analysis.

pHPT was biochemically diagnosed by the presence of inappropriately increased serum parathormone (PTH) and calcium levels (normal range 2.1–2.5 mmol/L), with normal or high 24 h urine calcium levels. PTH reference normal levels varied over time among different laboratories and were not comparable; thus, PTH values were scored as a ratio from multiples of the upper level of the normal range.

The suspicion of familial pHPT was confirmed when pHPT occurred in the index case and in at least one first-degree relative, with at least one confirmed pathological parathyroid gland at histology in each patient [5].

Genetic analysis was assessed after obtaining written consent during oncogenetic counselling. DNA was extracted from leukocytes and sequenced with next-generation sequencing, using a customized gene panel that included also *MEN1, RET, CDC73, CDKN1B, CASR, AP2S1, GNA11, CDKN1A, CDKN2B, CDKN2C* through both sequence analysis and copy number variation analysis. In the analysis, the mean sequencing depth was > 150 times; > 99% of the target nucleotides were covered with > 20× sequencing depth for all assays. The target nucleotides included all protein coding exons of the genes in the panels in addition to 20 base pairs inside each intron/exon boundary. Sequence variations in *GCM2* exons and exon–intron boundaries were all confirmed by Sanger sequencing. Variants are written according to Human Genome Variation Sequence nomenclature.

The pHPT surgical explorative strategy was categorized as ‘‘bilateral neck exploration’’ (aimed at the identification of parathyroid glands at both sides), or ‘‘focused exploration’’ (aimed at the unilateral identification of a single gland, according to the results of preoperative imaging).

Subtotal parathyroidectomy was defined as the removal of at least three glands, while selective parathyroidectomy referred to the removal of a single gland.

The histological diagnosis was recorded as performed soon after surgery and reviewed based on the 2022 WHO criteria [6].

pHPT biochemical cure was defined as the presence of postoperative normalization of serum calcium and appropriate PTH levels for at least 6 months after parathyroidectomy; persistent disease as pHPT occurring within 6 months after parathyroidectomy; recurrent disease as pHPT occurring after surgery followed by cure for at least 6 months [3].

Uniglandular involvement was confirmed by biochemical cure at the last follow-up after removal of a single gland. Multiglandular involvement was defined by the presence of pathology of more than one removed parathyroid gland, or by persistent or recurrent pHPT after excision of a single affected gland.

A literature search for papers focusing on *GCM2*-related pHPT was conducted using MEDLINE; keywords and medical subject headings used and combined by Boolean operators were: (‘*GCM2*’) and (‘hyperparathyroidism’) and (‘parathyroidectomy’ or ‘surgery’). A manual search of references in the eligible papers was also performed to identify additional pertinent articles. Only data concerning cases undergoing surgery were extracted. The search was limited to papers based on human studies, written in English and published between January 1980 and September 2024.

The study was performed in accordance with the guidelines in the Declaration of Helsinki and was approved by the Institution Review Boards.

## Results

The results of the study including 17 index cases of pHPT carrying germline *GCM2* variants are reported in the Table 1.

**Table 1:** Demographics, genetic, biochemical and clinical features, surgical strategy, pathology and outcome of surgery in 17 patients undergoing surgery for *GCM2*-related primary hyperparathyroidism

Patient ID	Gender	Age (years)	*GCM2* variant	Calcemia (mmol/L)	PTH ratio	Familial history of pHPT	Associated disease	Surgical strategy (initial procedure)	Pathology	Outcome	Parathyroid involvement	Follow-up (months)
Padua 1	M	49	c.1181 A>C, p.(Tyr394Ser)	3.8	4.39	Absent	Ewing’s sarcoma; uterine sarcoma; breast carcinoma, osteoporosis	BNE/ST-PTX	Multiple adenomas + atypical adenoma	Cure	MGD	60
Padua 2	F	62	c.1181 A>C, p.(Tyr394Ser)	2.8	2.87	Absent	Osteoporosis; urolithiasis	BNE/ST-PTX	Multiple adenomas	Cure	MGD	36
Padua 3	F	71	c.1181 A>C, (p.Tyr394Ser)	2.69	2	Absent	Osteoporosis, urolithiasis	FE/Se-PTX	Multiple adenomas	Persistent pHPT	MGD	130
Padua 4	F	72	c.1157 C>G, p.(Thr386Ser)	2.8	1.59	Absent	Osteoporosis, urolithiasis	BNE/ST-PTX	Multiple adenomas	Cure	MGD	60
Padua 5	F	52	c.1181 A>C, p.(Tyr394Ser)	3.44	6.31	Present	Osteoporosis	BNE/ST-PTX	Multiple adenomas	Cure	MGD	54
Padua 6	M	58	c.1166 T>C, p.(Val389 Ala)	2.9	5.39	Present	Osteoporosis, urolithiasis	BNE/ST-PTX	Multiple adenomas + carcinoma	Cure	MGD	7
Padua 7	F	51	c.1181 A>C, p.(Tyr394Ser)	2.89	NA	Present	Osteoporosis, urolithiasis	BNE/ST-PTX	Multiple adenomas	Cure	MGD	48
Padua 8	M	57	c.1181 A>C, p.(Tyr394Ser)	2.9	NA	Present		NA	Adenoma	NA	NA	NA
Padua 9	M	67	c.1166 T>C, p.(Val389 Ala)	2.94	5.21	Absent	Adrenal adenoma; Thyroid carcinoma; osteoporosis	NA	Atypical adenoma	NA	NA	NA
Padua 10	F	58	c.1185 dupG, p.(Pro396 Alafs* 8)	2.78	NA	Absent		NA	Adenoma	NA	NA	NA
Padua 11	M	70	c.1184 A>C, p.(Tyr394Ser)	2.8	NA	Absent	Prostate carcinoma	NA	NA	NA	NA	NA
Nancy 1	F	36	c.1010 T>C, p.(Leu337Pro)	2.67	1.31	Absent		FE/Se-PTX	Multiple adenomas	Persistent pHPT	MGD	39
Nancy 2	M	82	c.1181 A>C, p.(Tyr394Ser)	2.81	1.62	Present	Prostate carcinoma	FE/Se-PTX	Single adenoma	Cure	UGD	34
Nancy 3	F	32	c.1181 A>C, p.(Tyr394Ser)	3.23	2.75	Absent	Leukaemia	FE/Se-PTX	Multiple adenomas	Cure (Recurrent pHPT after 48 months and subsequent Persistent pHPT instead of reoperations	MGD (metachronous)	216
Nancy 4	F	33	c.844 T>G, p.(Tyr282 Asp)	2.7	2	Absent		FE/Se-PTX	Single adenoma	Cure	UGD	18
Nancy 5	F	57	c.1181 A>C, p.(Tyr394Ser)	3.03	2.61	Present	Breast carcinoma	FE/Se-PTX	Single adenoma	Cure	UGD	12
Nancy 6	F	40	c.844 T>G, p.(Tyr282 Asp)	2.77	3.51	Absent		FE/Se-PTX	Multiple adenomas	Persistent pHPT	MGD	102

*GCM2* variants are expressed as nucleotide, protein variations. Calcemia: normal value 2.1–2.5 mmol/L

*M* male, *F* female, *NA* not available, *BNE* bilateral neck exploration, *FE* focused exploration, *ST-PTX* subtotal parathyroidectomy, *Se-PTX* selective parathyroidectomy, *UGD* uniglandular parathyroid involvement, *MGD* multiglandular parathyroid involvement

The most common *GCM2* pathogenetic variant was c.1181 A>C p.(Tyr394Ser), observed in 10 out of 17 patients. *GCM2*-related pHPT affected both sexes with a slight predominance in females (Female/Male ratio = 1.83). The disease was diagnosed at a median age of 57 years (range 32–82). Preoperative median calcemia was 2.81 mmol/L (range 2.67–3.8), with a PTH ratio of 2.75 (range 1.31–6.31). Osteoporosis and urolithiasis were present in most patients; in some cases, prostate and breast carcinoma, bone Ewing’s and uterine sarcoma were also detected.

Family history of pHPT was absent in 11 out of 17 cases (65%). Pathology was available in 16 cases, including parathyroid adenomas (*n* = 13); atypical adenomas (*n* = 2) and parathyroid carcinoma (*n* = 1).

Complete clinical, surgical, outcome and follow-up data were available for 13 patients. At initial surgery, bilateral neck exploration with subtotal parathyroidectomy was performed in 6 out of 13 (46%) patients, while focused exploration with selective parathyroidectomy was performed in the remaining 7 (54%) cases. All 6 patients undergoing subtotal parathyroidectomy after bilateral neck exploration were cured and remained disease-free at a median follow-up of 51 months (range 7–60). When considering the 7 patients undergoing focused exploration with selective single parathyroidectomy, 3 had persistent pHPT; in one case (a 32-year-old patient) a recurrent pHPT occurred after a disease-free interval of 48 months following initial curative surgery, requiring subsequent unsuccessful reoperations; the remaining 3 patients were disease-free at a follow-up of 12, 18 and 34 months, at the age of 57, 33, and 82 years, respectively. Thus, at an overall prolonged follow-up (median 48 months, range 7–216) multiglandular involvement was found in 10 out of 13 patients (77%) at a median age of 51.5 years (range 32–72). Multiglandular involvement occurred synchronously in 9 patients while metachronously in one patient.

The literature search showed 12 studies including data on 137 *GCM2*-related pHPT operated patients, with a female/male ratio 1.51 and median age at surgery of 54 years (range 13–82). The most frequently detected genetic variation was c.1181 A>C p.(Tyr394Ser) (37% of cases). Atypical or malignant parathyroid lesions were found at histology in 14%; multiglandular involvement in 76% of cases; bilateral neck explorations were performed in only 24% of cases; the rate of overall postoperative cure was limited to 48% (Table 2).

**Table 2:** Literature review of studies reporting clinical data of patients undergoing surgery for *GCM2*-related primary hyperparathyroidism

Author (year)	Patients (*n* =)	Gender (F/M)	Age (median; range) (years)	*GCM2* variant	Calcemia (median; range) (mmol/L)	Associated disease	Surgical strategy^a^	Pathology	Parathyroid involvement	Outcome
Guan [4] (2016)	19	13/6	56 (25–79)	p.Asn315 Asp (*n* = 1); p.Gly203Ser + p.Ile227 Val (*n* = 1); p.Gln252Glu + p.Leu379Gln (*n* = 4); p.Tyr394Ser (*n* = 13)	2.8 (2.5–3.7)	Breast cancer (*n* = 1); colorectal cancer (*n* = 1); lung cancer (*n* = 1); pancreatic cancer (*n* = 1); ovarian cancer (*n* = 1)	FE/Se-PTX (*n* = 8); BNEST-PTX (*n* = 11)	Cancer (*n* = 1); NA (*n* = 18)	UGD (*n* = 2); MGD (*n* = 17)	Persistent pHPT (*n* = 9); NA (*n* = 10)
Guan [10] (2017)	18	9/9	60 (13–78)	p.Gln251Glu (*n* = 1); p.Tyr394Ser (*n* = 17)	2.7 (2.51–3.25)	Hodgkin’s lymphoma (*n* = 1); prolactinoma (*n* = 1); squamous cell cancer (*n* = 1)	FE/Se-PTX (*n* = 17); BNE/ST-PTX (*n* = 1)	NA	UGD (*n* = 1); MGD (*n* = 17)	Cure (*n* = 1); persistent pHPT (*n* = 13); recurrent pHPT (*n* = 1); NA (*n* = 3)
El Lakis [11] (2018)	18	12/6	54 (NA)	NA	2.85 (NA)	NA	BNE/ST-PTX (*n* = 14); BNE/ Se-PTX (*n* = 4)	Cancer (*n* = 1)	UGD (*n* = 4); MGD (*n* = 14)	Cure (*n* = 15); persistent pHPT (*n* = 3)
Cetani [5] (2020)	5	4/1	55 (27–62)	p.Gly203Ser (*n* = 2); p.Tyr394Ser (*n* = 3)	2.63 (2.55–2.67)	NA	FE/Se-PTX (*n* = 4); BNE/ST-PTX (*n* = 1)	Hyperplasia (*n* = 3); adenoma (*n* = 2)	UGD (*n* = 3); MGD (*n* = 2)	Cure (*n* = 3); persistent pHPT (*n* = 2)
Coppin [9] (2020)	47	29/18	47 (13–82)	p.Arg47 Cys + p.Gln330Leu (*n* = 1); p.Arg270Lys (*n* = 1); p.Arg406Gin (*n* = 1); p.Asp107 Asn (*n* = 1); p.Gln330Leu (*n* = 1); p.Val12Glu (*n* = 1); p.Lys56 Asn + p.Asp53 Asn (*n* = 2); p.Tyr394Ser (*n* = 3); p.Asn315 Asp (*n* = 5); p.Tyr282 Asp (*n* = 30); NA (*n* = 1)	2.78 (2.53–4.31)	Gastrinoma (*n* = 1); pancreatic neuroendocrine tumor (*n* = 1); pituitary adenoma (*n* = 1)	NA	Hyperplasia (*n* = 9); adenoma (*n* = 17); hyperplasia + adenoma (*n* = 1); NA (*n* = 1)	NA	NA
Song [7] (2020)	10	5/6	NA	p.Ala9 Ala (*n* = 1); p.Pro433Pro (*n* = 1); p.Tyr416 Cis (*n* = 1); p.Val382Met (*n* = 1); p.Lys388Glu (*n* = 2); p.Ser62Ser (*n* = 3)	NA	–	FE/Se-PTX (*n* = 9); BNE/ST-PTX (*n* = 1)	Hyperplasia (*n* = 1); adenoma (*n* = 7); cancer (*n* = 3); atypical adenoma (*n* = 1)	UGD (*n* = 9); MGD (*n* = 1)	Cure (*n* = 9); persistent pHPT (*n* = 1)
García-Castaño [12] (2021)	1	1/1	67 (NA)	p.Val382Met (*n* = 1)	2.55	NA	BN/ST-PTX (*n* = 1)		MGD (*n* = 1)	Persistent pHPT (*n* = 1)
Vincze [13] (2021)	9	NA	NA	p.Tyr394Ser (*n* = 5); p.Tyr282 Asp (*n* = 4)	NA	NA	NA	NA	NA	NA
Canaff [14] (2022)	5	3/2	56.5 (29–90)	p.Tyr386Ser (*n* = 2); p.Ile383Met (*n* = 3)	3.24 (2.57–3.7)	NA	FE/Se-PTX (*n* = 2); BNE/ST-PTX (*n* = 3)	Hyperplasia (*n* = 3); adenoma (*n* = 2); cancer (*n* = 2)	MGD (*n* = 5)	Cure (*n* = 3); persistent pHPT (*n* = 2)
Park [15] (2022)	1	1/0	27	p.Lys388Glu (*n* = 1)	2.6	NA	NA	NA	NA	NA
Tolkin [16] (2023)	2	0/2		p. Tyr394Ser (*n* = 2)	2.88 (2.7–3.07)	–	NA	Hyperplasia (*n* = 2)	MGD (*n* = 1); NA (*n* = 1)	NA
Szalat [17] (2023)	2	1/1	45 (19–71)	p.Tyr394Ser (*n* = 2)	2.82 (2.65–2.99)	–	FE/Se-PTX (*n* = 2)	Hyperplasia (*n* = 1), NA (*n* = 1)	MGD (*n* = 2)	Persistent pHPT (*n* = 2)
TOTAL	137	77/51	54 (13–82)	p.Ala9 Ala (*n* = 1); p.Arg270Lys (*n* = 1); p.Arg406Gln (*n* = 1); p.Arg47 Cys + p.Gln330Leu (*n* = 1); p.Asp107 Asn (*n* = 1); p.Gln251Glu (*n* = 1); p.Gln330Leu (*n* = 1); p.Gly203Ser + p.Ile227 Val (*n* = 1); p.Pro433Pro (*n* = 1); p.Ser193Ser (*n* = 1); p.Tyr416 Cis (*n* = 1); p.Val12Glu (*n* = 1); p.Gly203Ser (*n* = 2); p.Lys56 Asn + p.Asp53 Asn (*n* = 2); p.Tyr386Ser (*n* = 2); p.Val382Met (*n* = 2); p.Ile383Met (*n* = 3); p.Lys388Glu (*n* = 3); p.Ser62Ser (*n* = 3); p.Gln252Glu + p.Leu379Gln (*n* = 4); p.Tyr282 Asp (*n* = 4); p.Asn315 Asp (*n* = 6); p.Tyr282 Asp (*n* = 31); p.Tyr394Ser (*n* = 44); NA (*n* = 1)	2.75 (2.5–4.31)	Breast cancer (*n* = 1); colorectal cancer (*n* = 1); gastrinoma (*n* = 1); Hodgkin's lymphoma (*n* = 1); lung cancer (*n* = 1); pancreatic cancer (*n* = 1); ovarian cancer (*n* = 1); pancreatic neuroendocrine tumor (*n* = 1); pituitary adenoma (*n* = 1); prolactinoma (*n* = 1); squamous cell cancer (*n* = 1)	FE/Se-PTX (*n* = 42); BNE/ST-PTX (*n* = 18)	Hyperplasia (*n* = 19); Adenoma (*n* = 28); hyperplasia + adenoma (*n* = 2); cancer (*n* = 7); atypical adenoma (*n* = 1)	UGD (*n* = 19); MGD (*n* = 60)	Cure (*n* = 31); persistent pHPT (*n* = 33); recurrent pHPT (*n* = 1)

GCM2 variants are expressed as protein variations

*M* male, *F* female, *NA* not available, *BNE* bilateral neck exploration, *FE* focused exploration, *ST-PTX* subtotal parathyroidectomy, *Se-PTX* selective parathyroidectomy, *UGD* uniglandular parathyroid involvement, *MGD* multiglandular parathyroid involvement

^a^At initial surgical procedure

## Discussion

FIHPT is a rare (though likely underdiagnosed) variant of hereditary pHPT, and may be caused by several genes. It can occur as an incomplete form of classic syndromic pHPT due to incomplete expression of *MEN1, CDC73,* and cyclin-dependent kinase inhibitor (*CDKN*) genes. For this reason, a diagnosis of genuine FIHPT can be made after excluding these potential genetic variants. However, even rarer (and still largely unknown) genes may also cause FIHPT [1, 2].

In 2016, pathogenetic variants in *GCM2* have been identified as potential novel causes of FIHPT [4]. In fact, germline *GCM2* mutations have been reported in 18% of cases of FIHPT [4] and in 1.3% of all hereditary pHPT in a large Chinese series [7]. Moreover, somatic *GCM2* activating mutations have been also reported in 6.57% of sporadic parathyroid adenomas [8].

The *GCM2* gene is located on human chromosome 6p24.2; it encodes a 506 amino acid protein composed of several functional domains: a DNA-binding domain, 2 transactivation domains, a C-terminal conserved inhibitory domain and a nuclear localization signal [9]. In mammals, it is exclusively expressed in the parathyroid tissue and acts as a master regulator of parathyroid glands development. It activates PTH transcription in cooperation with other transcription factors such as GATA3 and MAF Beta [9], since the PTH promoter contains a consensus GCM-binding site, and *GCM2* is capable of activating the PTH promoter in reporter assays [4]. In mice, a loss of *GCM2* results in the absence of parathyroid glands development; in humans, germline frameshift, nonsense, and missense mutations leading to *GCM2* inactivation can cause a variant of hereditary hypoparathyroidism; in contrast, activating germline *GCM2* variants may cause hereditary pHPT, suggesting that *GCM2* acts as a parathyroid proto-oncogene [4, 9].

The present study reports 17 new index cases of operated patients carrying germline variants responsible for *GCM2*-related pHPT, representing one of the largest available series, to be added to the previously published 137 cases, as reported at the English-language literature review focusing on germline *GCM2* mutated patients undergoing surgery for pHPT [4, 5, 7, 9–17].

*GCM2*-related pHPT most likely occurs as an isolated variant (FIHPT) because *GCM2*-activating mutations are not expected to cause the non-parathyroid traits commonly seen in hereditary syndromic pHPT, such as those associated to MEN1 - 4 or HPT-JT Syndrome, given the highly restricted expression pattern of *GCM2* in parathyroid tissue [4]. However, the present series also showed the association with other neoplasms, including both common tumors in the general population (prostate and breast carcinomas), and rarer variants (Ewing’s and uterine sarcomas). Similar results have been found in literature, since both common (including colorectal, ovarian and breast cancers, adrenal and thyroid neoplasms, Hodgkin’s lymphoma and chronic lymphocytic leukemia) and less common neoplasms (such as gastrinomas, hypophyseal, and other pancreatic neuroendocrine tumors) [4, 5, 9, 10] have been documented. Although some of these associated tumors are also found in other hereditary syndromic pHPT variants, the role of *GCM2* gene in this setting has not been studied.

The most frequent germline variant identified in the present series was c.1181 A>C p.(Tyr394Ser) (59% of cases), which is also the most frequently reported genetic variant in literature (37% of cases) [4, 5, 9, 10, 13, 16, 17]. *GCM2* gain-of-function mutations are mostly located in exon 5, which encodes the C-terminal conserved inhibitory domain of the protein, suggesting that this region may be a hot spot for pathogenic variants [4, 9].

*GCM2*-related pHPT may mimic sporadic pHPT due to demographic factors (such as sex and age at diagnosis), personal and familial history, and biochemical features. In contrast to the expectation of an equal sex distribution due to the autosomal inheritance of *GCM2*, the present series found a slight prevalence in females (female/male ratio 1.8), consistent with the literature which reports an overall female/male ratio of 1.51 (Table 2). This might be explained by interactions with other genetic or epigenetic factors; the apparently increased prevalence in females may be also due to selection bias, as index cases might have been diagnosed through calcium levels measurement, which are more frequently detected in postmenopausal women following screening programs. Therefore, the true prevalence should be assessed by analyzing the entire kindred of mutation carriers; unfortunately, these data were not available in the present study.

Hereditary pHPT typically presents at a younger age compared to sporadic form. *GCM2* mutation carriers in the present series were diagnosed and operated on at a median age of 57 years (range 32–82), consistent with the literature reports (median 54 years, range 13–90) (Table 2). Therefore, age at diagnosis and surgery in *GCM2*-related pHPT does not significantly differ from sporadic pHPT [11]. However, earlier diagnosis (at age 13 and 17 years) have been previously reported [9, 11], which may result from prospective screening for hypercalcemia following the identification of a family member. In any case, follow-up starting before the age of 20 has been suggested in *GCM2* mutation carriers [11].

The present study confirmed that familial history of pHPT was absent at initial diagnosis in the majority of *GCM2* mutation carriers (65%), thus mimicking an apparently sporadic disease. Apparently sporadic presentation of hereditary pHPT at advanced age is not uncommon because the disease can remain asymptomatic over many years and thus underdiagnosed. Additionally, knowledge of relatives affected by pHPT is not always available; and incomplete penetrance and variable expressivity of *GCM2* mutations should be taken in account [1, 9]. In the Coppin series, pHPT was present in only 3 out of 7 carriers of the pathogenic variant c.1181 A>C, p.(Tyr394Ser), while the remaining 4 carriers (aged 80, 58, 34 and 33, respectively) remained disease-free [9]. This suggest that *GCM2* mutations have lower penetrance compared to other pathogenic genes responsible for MEN1, MEN4 or HPT-JT Syndrome [1, 9]. Interaction with *CASR* expression in parathyroid cells, as well as other factors and compensatory mechanisms, may explain the incomplete expressivity and penetrance of *GCM2* variants [9].

Furthermore, the median calcemia levels at surgery in the present series (2.81 mmol/L, range 2.67–3.8) were similar to those reported in the literature review (2.77 mmol/L range 2.5–4.3) (Table 2), which is not significantly different from sporadic pHPT [11]. Earlier published series described *GCM2*-related pHPT as a severe variant with very high calcemia and PTH levels series [4, 10, 11]; however, more recent series have found a prevalence of mild hypercalcemia, even with low urinary calcium excretion fraction [17]. In the present series, severe pHPT with very high calcium and PTH levels occurred in 19% of cases, due to parathyroid atypical adenoma (2 cases) and carcinoma (1 case), as it is typically seen in HPT-JT Syndrome [1–3] while very rarely in sporadic variants. Similar findings are evident in the literature review, where parathyroid malignancies have been reported in 14% of cases [4, 10, 11]. However, the higher rate of malignant and atypical parathyroid tumors in *GCM2* mutation carriers is based on very few cases; thus, further studies and surveillance of *GCM2* mutation carriers’ kindred are needed to confirm this association [11].

In contrast to the common finding in sporadic pHPT, multiglandular parathyroid involvement is very frequent in *GCM2*-related pHPT. This results in a lower rate of cure following focused approaches and selective parathyroid removal, as well as increased rate of persistent and recurrent pHPT, leading to multiple reoperations and subsequent increased morbidity [4, 5, 7, 10, 11, 14, 17].

In the present series, multiglandular involvement occurred in 77% of cases and was yet present at the time of surgery in 9 out of 10 patients, with a median age of 51.5 years (range 32–72). In a 32-year-old patient with initial single gland involvement, additional parathyroid adenomas developed metachronously, causing recurrent disease 4 years after the initial surgery. Therefore, single gland involvement was observed in only 3 patients, aged 33, 57 and 82 years. Therefore, after excluding the oldest patient, recurrent pHPT is likely to occur when prolonging the follow-up. The best results were achieved with bilateral neck exploration and subtotal parathyroidectomy, which resulted in cure for all cases, with disease-free status at a prolonged follow-up. In contrast, focused explorations and selective parathyroidectomies failed in 43% of cases, causing a persistent pHPT. Similar results are evident at literature review: multiglandular involvement occurred in 76%; focused approaches and selective parathyroidectomy were performed in 70% of cases, and as a consequence, cure was achieved only in a minority of cases, with an overall rate of persistent pHPT exceeding 50% [4, 5, 7, 9–17].

For these reasons, a routine more aggressive approach including bilateral neck exploration and subtotal parathyroidectomy (similar to the approach used in MEN1) [1] should be the preferred surgical strategy to achieve long-term cure and prevent failures of selective parathyroidectomy. This is particularly relevant in light of suspected increased risk of malignancies, especially in case with very high calcium and PTH levels [7, 11].

Thus, the knowledge of carrier status in patients with germline *GCM2*-activating mutations should be available whenever possible before the initial operation. This strategy not only could provide the best chance for definitive treatment but also minimize the need for reoperations and associated morbidity [11].

The current study has several limitations. The retrospective study design limits our ability to fully ascertain disease characteristics, penetrance and expressivity of the gene, particularly due to the lack of data on relatives of operated patients and the entire kindred. Furthermore, the interpretation of the sequence variations identified in *GCM2* is challenging, as the pathogenetic role of some variants remains still controversial, because of the lack of functional studies able to confirm the consequences of the variants [9]. Additionally, the incomplete penetrance of pathogenic variants in the absence of a complete clinical evaluation of mutation carriers in each kindred, along with the possibility to have “healthy” mutation carriers or patients with a very late disease onset, prevents any definitive conclusion about the management of this condition [9].

## Conclusions

*GCM2* germline mutations may represent a cause of hereditary pHPT, although they can mimic sporadic variants due to the absence of a familial history and the late onset of the disease, likely resulting from incomplete penetrance and expression. The main feature is multiglandular involvement, which necessitates bilateral neck exploration and subtotal parathyroidectomy to achieve long-term cure. Focused exploration and selective single gland removal may lead to persistent pHPT, or recurrent pHPT at long-term follow-up. Multiple parathyroid adenomas are the most frequent finding at pathology; atypical adenomas and carcinomas may be also found. Therefore, *GCM2* sequencing should be included in the systematic genetic screening of pHPT patients with a familial history of pHPT, multiglandular parathyroid involvement, or cases with persistent or recurrent pHPT, as well as atypical or malignant involvement. This is crucial for identifying mutation carriers, who require tailored management with more aggressive and extensive approach.

## Data Availability

The data that support the finding of this study are available on request from the corresponding author.
